# Identification of four snoRNAs (SNORD16, SNORA73B, SCARNA4, and SNORD49B) as novel non-invasive biomarkers for diagnosis of breast cancer

**DOI:** 10.1186/s12935-024-03237-0

**Published:** 2024-02-04

**Authors:** Xiao Li, Xuan Zhao, Li Xie, Xingguo Song, Xianrang Song

**Affiliations:** 1 Department of Clinical Laboratory, Shandong Cancer Hospital and Institute, Shandong First Medical University, Shandong Academy of Medical Sciences, 440 Ji-Yan Road, Jinan, 250117 Shandong Province China; 2 Shandong Provincial Key Laboratory of Radiation Oncology, Shandong Cancer Hospital and Institute, Shandong First Medical University, Shandong Academy of Medical Sciences, Jinan, Shandong China

**Keywords:** snoRNAs, SNORD16, SNORA73B, SCARNA4, SNORD49B, Biomarker, Breast cancer

## Abstract

**Background:**

Emerging data point to the critical role of snoRNA in the emergence of different types of cancer, but scarcely in breast cancer (BC). This study aimed to clarify the differential expressions and potential diagnostic value of SNORD16, SNORA73B, SCARNA4, and SNORD49B in BC.

**Methods:**

We screened differential snoRNAs in BC tissues and adjacent tissues through SNORic datasets, and then we further verified them in the plasma of BC patients and healthy volunteers by quantitative polymerase chain reaction (qPCR).

**Results:**

These four snoRNAs: SNORD16, SNORA73B, SCARNA4, and SNORD49B were considerably more abundant in cancerous tissues than in neighboring tissues in the TCGA database. Their plasma levels were also higher in BC and early-stage BC patients when compared to healthy controls. Furthermore, the ROC curve demonstrated that BC (AUC = 0.7521) and early-stage BC (AUC = 0.7305) might be successfully distinguished from healthy people by SNORD16, SNORA73B, SCARNA4, and SNORD49B.

**Conclusion:**

Plasma snoRNAs: SNORD16, SNORA73B, SCARNA4, and SNORD49B were upregulated in BC and early-stage BC and can be used as potential diagnostic markers for BC and early-stage BC.

**Supplementary Information:**

The online version contains supplementary material available at 10.1186/s12935-024-03237-0.

## Introduction

Breast cancer (BC), the most frequent gynecological cancer, has surpassed lung cancer to become the most frequently diagnosed cancer in the world, underscoring the importance of global guidance for optimal treatment [1]. According to statistical data from the National Cancer Center, BC was the most diagnosed malignant neoplasm and the second leading cause of cancer mortality among Chinese females, accounting for 16.72% (306,000) of all new cancer cases. About 71,700 people died in BC, with a crude mortality of 10.62 (per 10^5^ population) in 2016 [2]. The good news is that the BC mortality is continuing to fall at a steady rate, which largely depends on advances in early detection and treatment [3]. Therefore, only early-stage BC patients benefit significantly from the continuous improvement of therapeutic strategies, concurrently, the sensitive and reliable biomarkers are urgently needed to assist in the early diagnostics of BC.

Small nucleolar RNAs (snoRNAs) are non-coding RNAs with 60–300 nt in length [4]. It is mainly divided into two categories: C/D box and H/ACA box snoRNAs, which mediate 2’-O-ribomethylation and pseudoacidification of ribosomal RNAs (rRNAs), respectively, as well as small Cajal body-specific RNAs (scaRNAs), a specific subset of snoRNAs accumulated in Cajal bodies which guide the post-transcriptional modification of splicing body RNAs [5–7]. In recent years, increasing evidence has demonstrated that snoRNAs play important roles in tumorigenesis and the development of BC [8, 9]. For example, a previous study demonstrated that snoRNAs’ elevated biogenesis is essential in BC; U22, U3, U8, U94, and U97 snoRNAs were significantly elevated in BC tissues [10], whereas other researchers had reported that SNORD50 [11, 12], U44/SNORD44, SNORD43, and SNORD48 [13] were down-regulated in BC. snoRNAs show significant differential expressions in normal and BC; measuring snoRNA levels could be useful for diagnosis and prognostic applications in BC [8].

Previous studies have proved snoRNAs are stably expressed in blood, plasma, urine, and other body fluids; thereby, they possess the potential to be cancer biomarkers. For example, SNORD33, SNORD66, and SNORD76 existed in a stable form and could be reliably measured in plasma, serving as the potential biomarkers for non-small cell lung cancer (NSCLC) [14]. In a recent study, our group identified a critical role of SNORD88C in NSCLC; it was upregulated in tissue and plasma and served as a non-invasive diagnostic biomarker, promoting NSCLC proliferation, invasion, and metastasis both in vivo and in vitro [15]. We also revealed a three-snoRNA signature: SNORD15A, SNORD35B, and SNORD60 were upregulated in the tissues and urinary sediment of renal cell carcinoma (RCC), serving as novel potential biomarkers for RCC diagnosis [16]. In the BC, SNORA7B was significantly upregulated, and patients with high SNORA7B expression had a worse prognosis [17]. The above list indicates that snoRNAs can serve as a potential diagnostic and prognostic biomarker for cancer.

In the present study, we screened out SNORD16, SNORA73B, SCARNA4, and SNORD49B based on the database and then performed further validation in cancerous and healthy plasma. Our data demonstrated these four snoRNAs were significantly increased in the plasma of BC patients and early-stage patients and possessed good diagnostic efficiency, suggesting plasma SNORD16, SNORA73B, SCARNA4, and SNORD49B maybe can act as a novel noninvasive biomarker for BC.

## Materials and methods

### Data source

SnoRNA gene expression data of BC and adjacent tissues were downloaded from the SNORic database (http://bioinfo.life.hust.edu.cn/SNORic). The clinical information of BC patients included above was obtained from TCGA (http://cancergenome.nih. gov). BRCA database. There are 104 healthy volunteers and 1077 BC patients were included in this study.

### Patients, healthy donors, and sample collection

In this research, plasma samples were collected from BC cancer patients admitted to Shandong Cancer Hospital and Institute from March 2022 to March 2023. Inclusion Criteria for BC patients: over 18 years old; primary BC without any anti-tumor treatment; no other endocrine, immune, or metabolic diseases. Exclusion Criteria: non-primary tumor; BC combined with other tumors; receiving any anti-tumor treatment; other endocrine, immune, or metabolic diseases besides BC. The TNM stage was determined based on the American Joint Committee on Cancer’s (AJCC) eighth edition. The detailed clinical information of the BC patients was listed in (Tables 1 and 2). Inclusion Criteria for healthy volunteers: over 18 years old; no current or past cancer diagnosis; excluding endocrine, immune, metabolic, or malignant diseases; healthy/free from known illness at all times of blood collection.

**Table 1:** Correlation between plasma snoRNAs expression and clinicopathologic characteristics of BC patients

Characteristics		SNORD16		SNORA73B		SCARNA4		SNORD49B	
		Case No.	*P* value	Case No.	*P* value	Case No.	*P* value	Case No.	*P* value
**Age**	≤ 51	122	0.8895	123	0.4033	122	0.8613	122	0.3928
	> 51	106		111		111		108	
**T stage**	Tis	9	0.0350*	9	0.3952	9	0.1684	8	0.0090*
	T1	64		68		68		65	
	T2	86		85		83		86	
	T3	11		11		11		11	
	T4	15		17		17		16	
	unknown	43		44		45		44	
**Lymph node metastasis**	N0	94	0.7340	95	0.4006	93	0.6994	94	0.6861
	N1	44		45		45		44	
	N2	28		30		30		28	
	N3	17		18		18		18	
	Unknown	45		46		47		46	
**Distant metastasis**	M0	171	0.6331	176	0.7222	174	0.7009	172	0.4625
	M1	12		12		12		12	
	Unknown	45		46		47		46	
**TNM stage**	Tis	8	0.0538	8	0.2053	8	0.2888	7	0.0180*
	I	42		43		43		43	
	II	76		76		74		75	
	III	42		46		46		44	
	IV	9		9		9		9	
	Unknown	51		52		53		52	
**ER**	-	68	0.1855	68	0.0681	66	0.3334	67	0.3058
	+	154		160		161		157	
	Unknown	6		6		6		6	
**PR**	-	94	0.0807	96	0.0130*	94	0.1888	95	0.1015
	+	127		131		132		128	
	Unknown	7		7		7		7	
**HER2**	-	150	0.2007	156	0.1128	154	0.3975	151	0.2009
	+	65		64		65		66	
	Unknown	13		14		14		13	
**Ki67**	-	52	0.0504	54	0.0507	52	0.1296	52	0.0720
	+	170		174		175		172	
	Unknown	6		6		6		6	
**Subtype**	Luminal	126	0.9394	132	0.5234	130	0.9405	128	0.9071
	HER2-enriched	51		50		50		52	
	Triple-negative	24		24		24		23	
	Unknown	27		28		29		27	

**Table 2:** Correlation between plasma snoRNAs expression and clinicopathologic characteristics of early-stage BC patients

Characteristics		SNORD16		SNORA73B		SCARNA4		SNORD49B	
		Case No.	*P* value	Case No.	*P* value	Case No.	*P* value	Case No.	*P* value
**Age**	≤ 51	65	0.6872	66	0.6830	65	0.7079	66	0.2119
	> 51	61		61		60		59	
**T stage**	Tis	8	0.0258*	8	0.1096	8	0.1389	7	0.0038*
	T1	53		55		55		54	
	T2	60		59		57		59	
	T3	5		5		5		5	
**Lymph node metastasis**	N0	88	0.4689	88	0.2685	86	0.4420	87	0.3159
	N1	38		39		39		38	
**TNM stage**	Tis	8	0.0180*	8	0.0376*	8	0.1020	7	0.0052*
	I	42		43		43		43	
	II	76		76		74		75	
**ER**	-	41	0.2434	40	0.4287	39	0.1063	39	0.0357*
	+	85		87		86		86	
**PR**	-	54	0.6593	53	0.8912	51	0.1058	52	0.0646
	+	72		74		74		73	
**HER2**	-	89	0.4923	89	0.2675	88	0.3720	88	0.4828
	+	32		32		31		32	
	Unknown	5		6		6		5	
**Ki67**	-	32	0.1004	32	0.1578	30	0.5481	31	0.6476
	+	94		95		95		94	
**Subtype**	Luminal	74	0.1595	74	0.1641	73	0.1344	74	0.2740
	HER2-enriched	28		28		27		28	
	Triple-negative	15		15		15		14	
	Unknown	9		10		10		9	

Peripheral blood was collected and stored in an EDTA tube and then centrifuged at 3000×g for 10 min at 4 °C to separate from the peripheral blood cells, followed by another 12,000×g centrifugation for 10 min at 4 °C to pellet any remaining cells. The supernatant was collected and stored at − 80 °C until use. Notably, since we performed the detections using the remaining samples after clinical laboratory tests which had been approved by the ethics committee, not all of them could cover the demand to detect all four snoRNAs. The sample size was listed in Table S1, in which samples from 212 BC patients and 203 healthy volunteers were subjected to the detection of all four snoRNAs.

### Isolation of exosomes and microvesicles

The separation of exosomes and microvesicles (MVs) generally included the following steps, as described above [18–21]. First, peripheral blood was centrifuged at 3000×g for 10 min at 4 °C to collect plasma, followed by another two centrifugations at 14,000×g for 35 min at 4 °C to obtain MV pellets. Simultaneously, the above MV-poor plasma underwent ultracentrifugation (Type 50.4 Ti Rotor; Beckman Coulter; CA, USA) at 100,000×g for 120 min at 4 °C to isolate exosomes.

### RNA extraction

Total plasma RNA was extracted using 750 ul of TRIzol® LS Reagent (Thermo Fisher, Carlsbad, CA, USA) per 250 ul of plasma according to the reagent instructions, while the extraction of total RNA from microcapsules and exocrine bodies was done with 500 ul of TRIzol Reagent (Thermo Fisher Scientific, Carlsbad, CA, US).

### Reverse transcription and quantification by real-time PCR

Reverse transcription was performed using the Mix-X miRNA First-Strand Synthesis Kit (Accurate Biotechnology, Hunan, China), and quantitative PCR was carried out using the SYBR Green Premix Pro Taq HS qPCR Kit reagent (Accurate Biotechnology, Human China) through the LC480 system (Roche, Basel, Switzerland). U6 acted as the internal reference [22]. The relative expressions of snoRNAs were evaluated by the comparative cycle threshold (CT) method: (ΔCT = CT^snoRNAs^-CT^U6^), as described previously [23]. The qPCR primers are listed in (Table 3).

**Table 3:** Primer sequences involved

Gene	Forward primer	Reverse primer
SNORD19B	TGGTTGAAATATGATGAGTGTAC	GAAATCAGAGTTGGATCTTGTAA
SNORA25	TGAGGCTGTGAAACCCAGAG	GCACCAAACATTAGGAGTGC
SNORA65	TCTCTGTTGGCTGGTGCAAT	TGCTTTCGGCACAGAGTCAT
SNORD15A	GGGGATGTTCTCTTTGCCCA	AGGGCTCTTTAAACTGTGCCA
SNORD2	ATGGCAATCATCTTTCGGGAC	CAAGTGATCAGCAATGAGTATTCT
SNORD12B	TTTTTCCCCGACAGATCGAC	GCTCAAGCTGGCATATCAGAC
SNORA64	TCGGCTCTGCATAGTTGCAC	TGCACCCCTCAAGGAAAGAG
SNORA70B	AGCCAATTAAGCCGACTGAGTTCC	GTCCCTTAGAGCAACCCATACAACC
SNORA24	TGTCAAGTGTGGCAGTCTCC	ATGGTGACAGCTTTGCTGGT
U76	GCCACAATGATGACAGTTTATTTGC	GCCTCAGTTAAGATAATGGTGGT
U44	GAGAATACTCATTGCTGATCACTTG	universal primer
SNORD5	TCAGATGATGAATTTAACTGTTCAACTG	AGTTTTAGTTCATGCCCGTTATCA
SNORA69	AGGTTGCAATTACAGTGCTTCA	CACGGCTTTTCTTTCAGCAGG
SNORD41	GGAAGTGATGACACCTGTGACT	CAGCCAGTACGAATACGCGA
SNORD16	TGCAATGATGTCGTAATTTGCGTC	TCGTCAACCTTCTGTACCAGCT
SNORA73B	AGGCTCTGTCCAAGTGGCATAGG	CGAGGCCCAGCTTCATCTTCAAC
SCARNA4	CTGGAGGACTAAGAAGGCTGA	GAAGGCTGCTCTCTCCAACC
SNORD49B	TGATACTTGTAATAGGAAGTGCCG	CAGATAGACATTAGACGTCGTCAG
U6	TGGAACGCTTCACGAATTTGCG	GGAACGATACAGAGAAGATTAGC

### Statistical analysis

Statistical analyses were performed using the SPSS software (Cary, NC, USA) and GraphPad Prism software 9.0 (San Diego, CA, USA). Data in (Tables 1 and 2) were shown in the form of interquartile spacing. The Kolmogorov-Smirnov test was used to test the normality of the data. If normal, the unpaired t-test was used for the analysis of two groups of data and a one-way analysis of variance (ANOVA) for the analysis of multiple groups. If not, Mann-Whitney U test was used for two groups of data and the Kruskal-Wallis test for multiple groups of analysis. Two-tailed *p* < 0.05 was defined as statistically significant.

## Results

### Identification of the aberrant snoRNAs in BC from the database

**Fig. 1:**
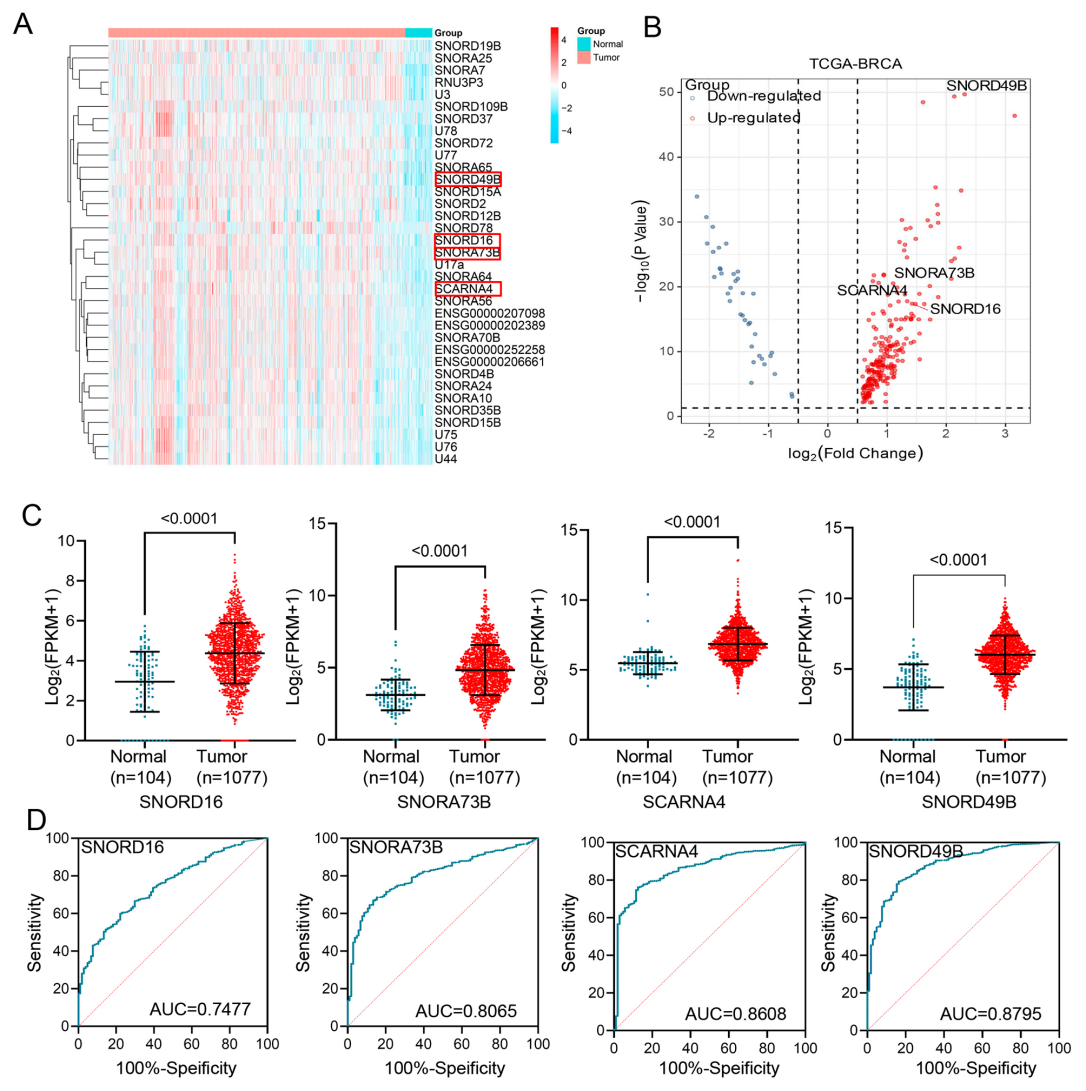
Identification of the aberrant snoRNAs in BC from the database. **(A-B)** Heat maps **(A)** and volcano plots **(B)** show the differential expressions of snoRNAs in BRCA tumor tissues (*N* = 1077) and adjacent tissues (*N* = 104), respectively. **(C-D)** The differential expression **(C)** and AUCs **(D)** of plasma snoRNAs SNORD16, SNORA73B, SCARNA4, and SNORD49B were analyzed in BRCA and adjacent tissues, respectively. BRCA: breast invasive carcinoma

In order to screen the differential snoRNAs, we downloaded the data of snoRNA expression profiles of 104 control tissues and 1077 tumor tissues from the TCGA and SNORic databases. Differential expressions of snoRNAs between BC patients and healthy donors were identified and presented in a heat map (Fig. 1A) and volcano map (Fig. 1B) according to the following criteria: an adjusted *p* value < 0.05 and an absolute log fold change > 2.00. Then, the above peer-selected snoRNAs were detected in the plasma from a small-size cohort, including 24 BC patients and 24 healthy volunteers, except those that expressed very low circulation or varied significantly in different individuals. Unexpectedly, most of them, including SNORD19B, SNORA25, SNORA65, SNORD15A, SNORD2, SNORD12B, SNORA64, SNORA70B, SNORA24, U76, U44, SNORD5, SNORA69, and SNORD41, were not significantly discrepant in plasma (Fig. S2), unlike those in tissues compared to healthy donors, implying an aberrant expression pattern in circulation. SNORD16, SNORA73B, SCARNA4, and SNORD49B were ultimately selected as candidate genes and further validated in a large sample due to their differential expressions between BC and the healthy group.

Subsequently, we analyzed the differential expressions of the above-mentioned four snoRNAs using the database. As shown in Fig. 1C, these four snoRNA levels were significantly increased in tumor tissues compared with normal tissues, and their respective areas under the curve (AUC) were 0.7477, 0.8065, 0.8608, and 0.8795. (Fig. 1D).

### Stable expressions of SNORD16, SNORA73B, SCARNA4, and SNORD49B in vesicle-free plasma

**Fig. 2:**
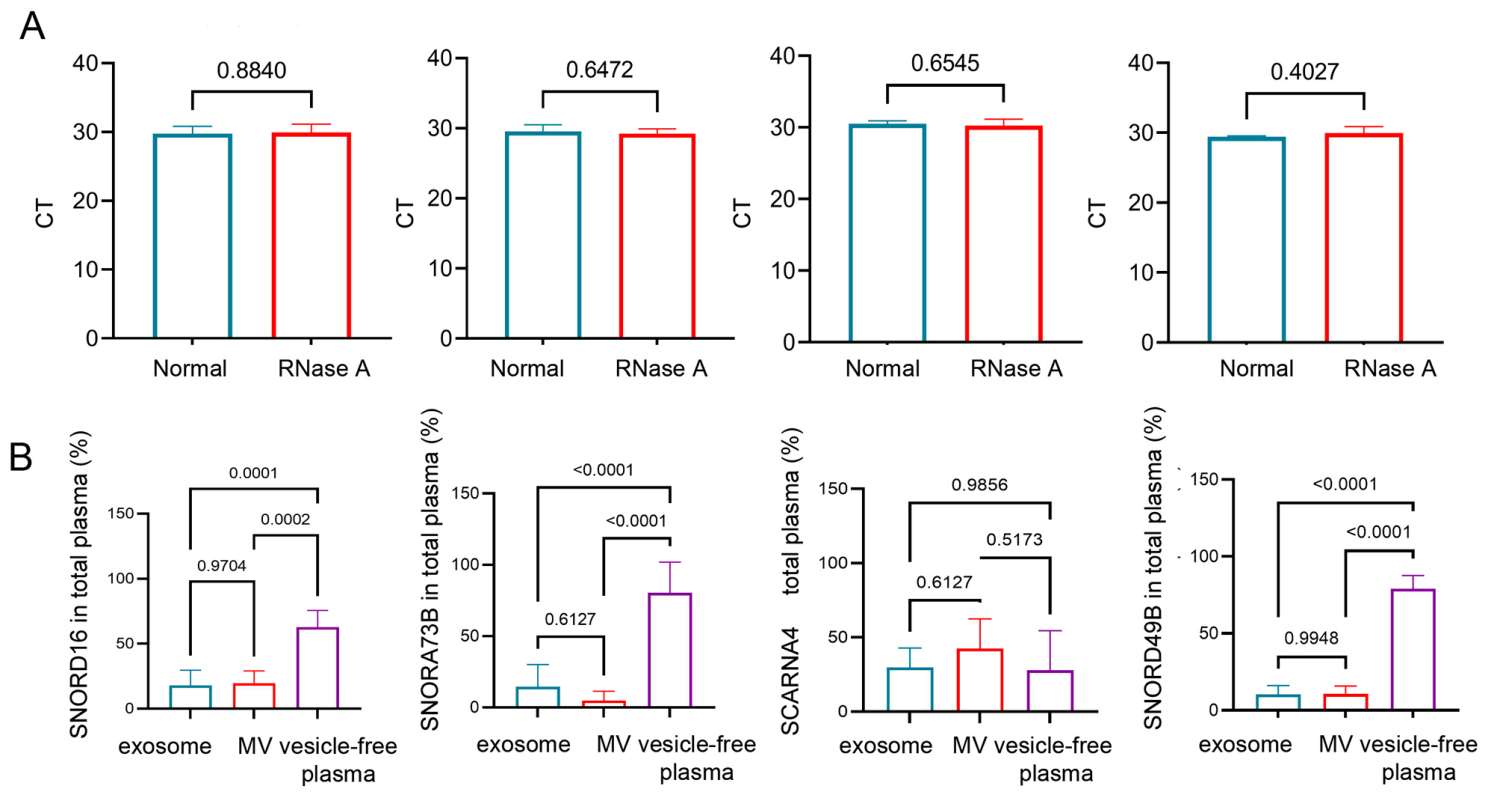
Stable expressions of SNORD16, SNORA73B, SCARNA4, and SNORD49B in vesicle-free plasma. **(A)** The expressions of SNORD16, SNORA73B, SCARNA4, and SNORD49B in plasma after RNase A treatment **(B)** The location of SNORD16, SNORA73B, SCARNA4, and SNORD49B in plasma. MV: microvesicle

We first tested whether these four snoRNAs we screened were stable in plasma treated with RNase A. As shown in Fig. 2A, RNase A failed to cause a significant change in the CT values of these four snoRNAs, indicating their stability in the circulation. Next, we explored the position in plasma by detecting their expressions in MVs, exosomes, and vesicle-free plasma. As shown in Fig. 2B, SNORD16, SNORA73B, and SNORD49B are mainly expressed in vesicle-free plasma other than in MVs and exosomes. Nevertheless, no significant difference in SCARNA4 expression was observed among MVs, exosomes, or vesicle-free plasma.

### Plasma snoRNAs: SNORD16, SNORA73B, SCARNA4, and SNORD49B as biomarkers for BC diagnosis

**Fig. 3:**
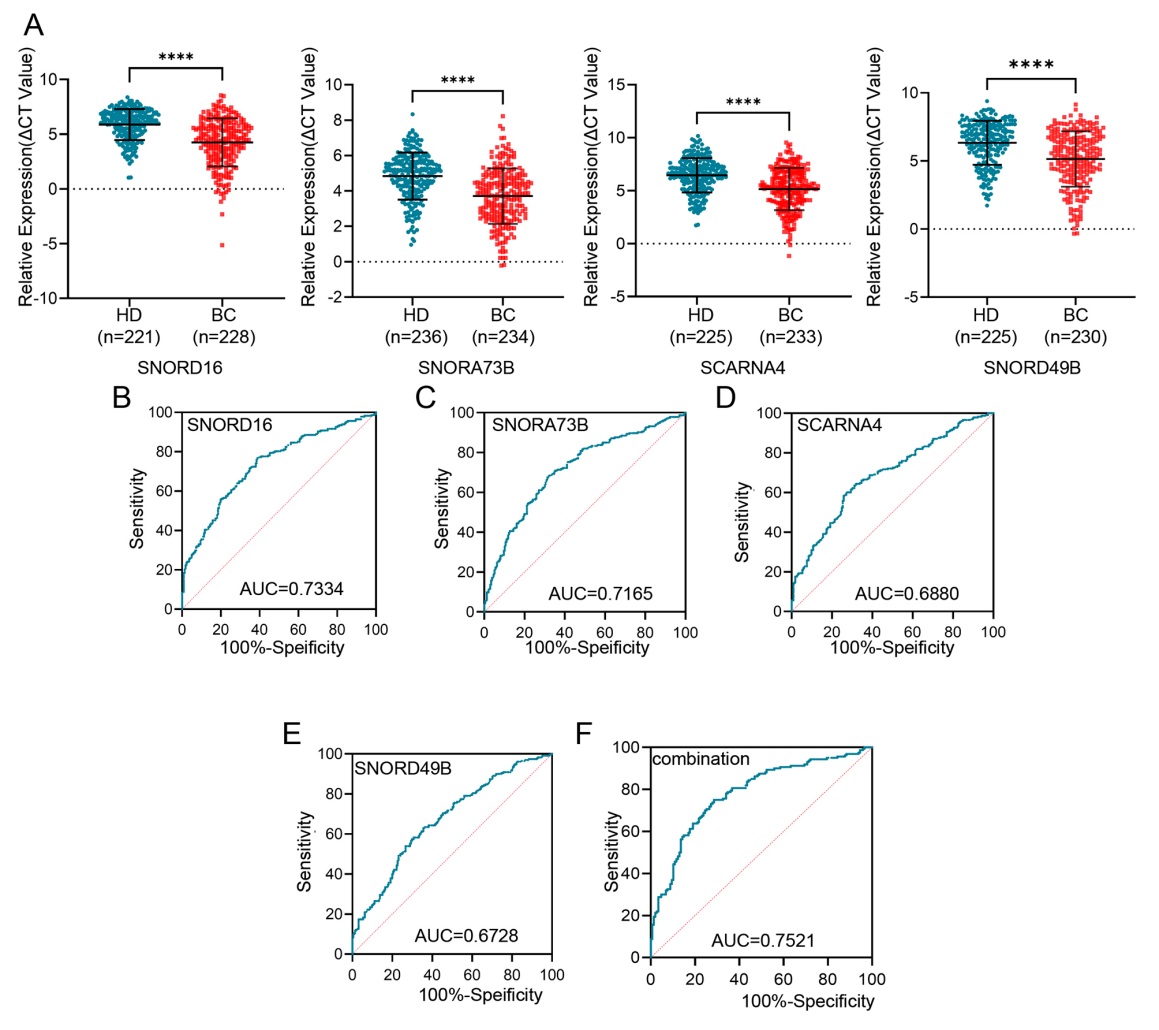
Plasma snoRNAs: SNORD16, SNORA73B, SCARNA4, and SNORD49B as biomarkers for BC diagnosis. **(A)** The differential expressions of plasma SNORD16, SNORA73B, SCARNA4, and SNORD49B were analyzed between BC patients and health donors. The AUCs of plasma SNORD16 **(B)**, SNORA73B **(C)**, SCARNA4 **(D)**, and SNORD49B **(E)** were 0.7334, 0.7165, 0.6880, and 0.6728, respectively. **(F)** The diagnostic performance for their combination demonstrated an AUC of 0.7521. ****P < 0.0001

Next, SNORD16, SNORA73B, SCARNA4, and SNORD49B were subjected to a large cohort with over 200 breast cancer patients and healthy volunteers to verify their diagnostic role for BC. As shown in Fig. 3A, plasma SNORD16, SNORA73B, SCARNA4, and SNORD49B were up-regulated in BC patients compared with those in the healthy, consistent with the results in the database. The association between snoRNA expression and different clinicopathological parameters in BC patients was also evaluated as listed in Table 1. SNORD16 was correlated with T stage (*p* = 0.0350); SNORA73B was correlated with progesterone receptor (PR) (0.0130); SNORD49B was correlated with the T stage (0.0090) and TNM stage (0.0180), whereas there were no statistically significant correlations with other clinical characteristics.

A receiver-operating characteristic (ROC) curve was computed to investigate the possibility of snoRNAs as a circulating diagnostic marker for BC. The expressions of SNORD16, SNORA73B, SCARNA4, and SNORD49B successfully discriminated BC patients from healthy volunteers (Fig. 3B-E), possessing AUCs of 0.7334 (Fig. 3B) with 76.75% sensitivity and 61.09% specificity, 0.7165 (Fig. 3C) with 68.38% sensitivity and 67.8% specificity, 0.6880 (Fig. 3D) with 58.37% sensitivity and 74.22% specificity, and 0.6728 (Fig. 3E) with 57.39% sensitivity and 70.22% specificity, respectively. When combined, the diagnostic efficacy of these four snoRNAs was 0.7521 (Fig. 3F) with 66.51% sensitivity and 74.38% specificity, indicating that plasma snoRNAs are promising noninvasive diagnostic biomarkers for BC.

### Plasma snoRNAs: SNORD16, SNORA73B, SCARNA4, and SNORD49B as biomarkers for early diagnosis of BC

**Fig. 4:**
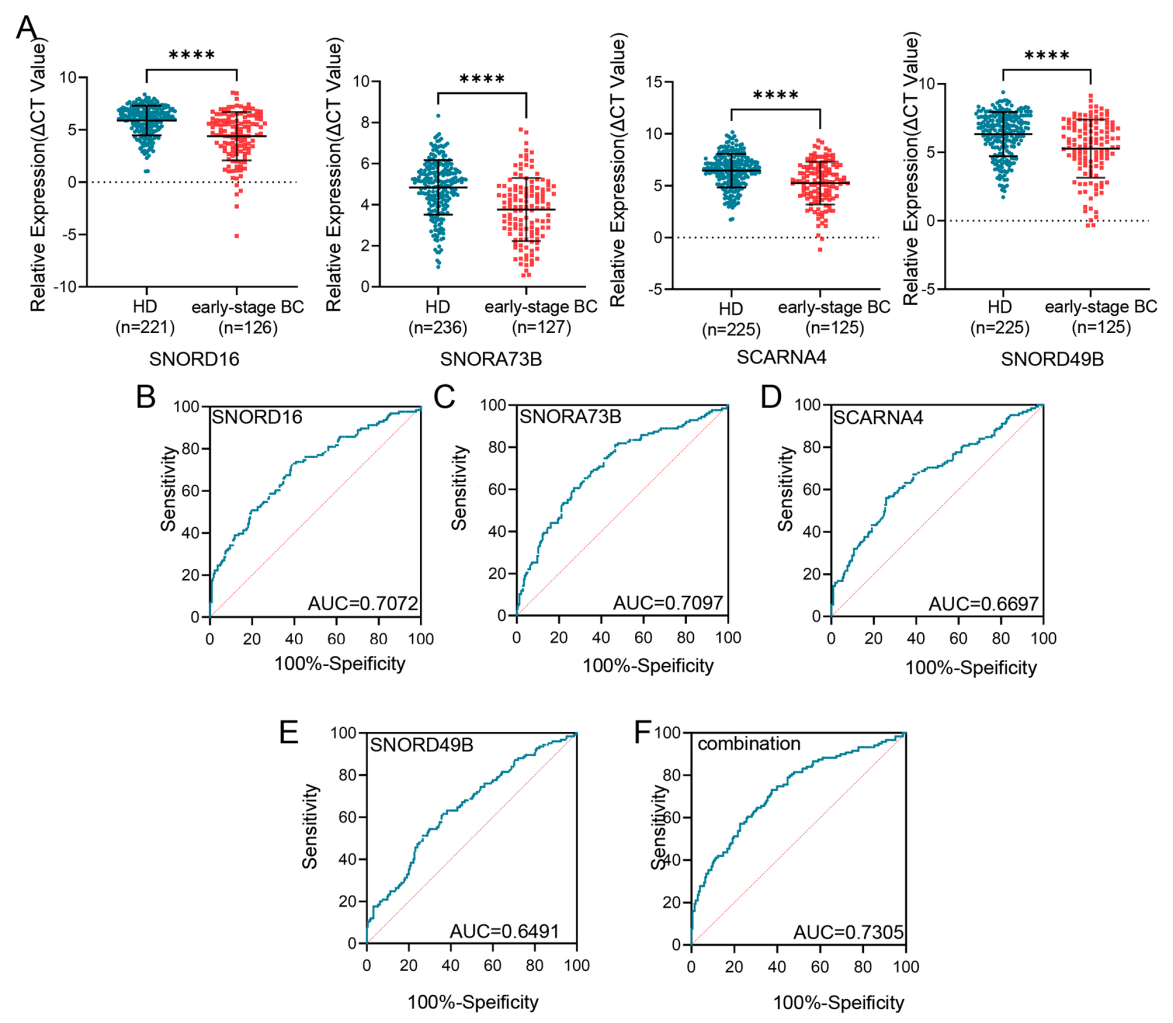
Plasma snoRNAs: SNORD16, SNORA73B, SCARNA4, and SNORD49B as biomarkers for early diagnosis of BC. **(A)** The differential expressions of plasma SNORD16, SNORA73B, SCARNA4, and SNORD49B were analyzed in early-stage BC patients compared with health donors. The AUCs of plasma SNORD16 (B), SNORA73B **(C)**, SCARNA4 **(D)**, and SNORD49B **(E)** were 0.7072, 0.7097, 0.6697, and 0.6491, respectively. **(F)** The diagnostic performance for their combination demonstrated an AUC of 0.7305. ****P < 0.0001

Furthermore, we analyzed the expression and diagnostic accuracy of these four plasma snoRNAs for early-stage BC patients (stages Tis + I + II). Consistently, the levels of these four were significantly higher in early-stage BC patients than in healthy donors (Fig. 4A). Meanwhile, Table 2 lists the results of the evaluation of the relationship between snoRNA expression and other clinicopathological characteristics in early-stage BC patients. SNORD16 was correlated with T stage (*p* = 0.0258) and TNM stage (0.0180); SNORA73B was correlated with TNM stage (0.0376); and SNORD49B was correlated with T stage (0.0038), TNM stage (0.0052), and ER status (0.0357). However, no other statistically significant correlations were observed.

Then, ROC curve analysis was constructed to show their diagnostic performance. The AUCs were 0.7072 (Fig. 4B) with 72.22% sensitivity and 61.09% specificity, 0.7097 (Fig. 4C) with 81.10% sensitivity and 53.39% specificity, 0.6697 (Fig. 4D) with 56.00% sensitivity and 74.22% specificity, and 0.6491 (Fig. 4E) with 60.80% sensitivity and 64.44% specificity for SNORD16, SNORA73B, SCARNA4, and SNORD49B, respectively. When the four snoRNAs were combined, the AUC increased to 0.7305 (Fig. 4F) with a sensitivity of 73.11% and a specificity of 62.56%, suggesting that these four snoRNAs may be useful as early-stage BC diagnostic biomarkers.

### Plasma snoRNAs enhance the diagnostic accuracy of CEA for BC

**Fig. 5:**
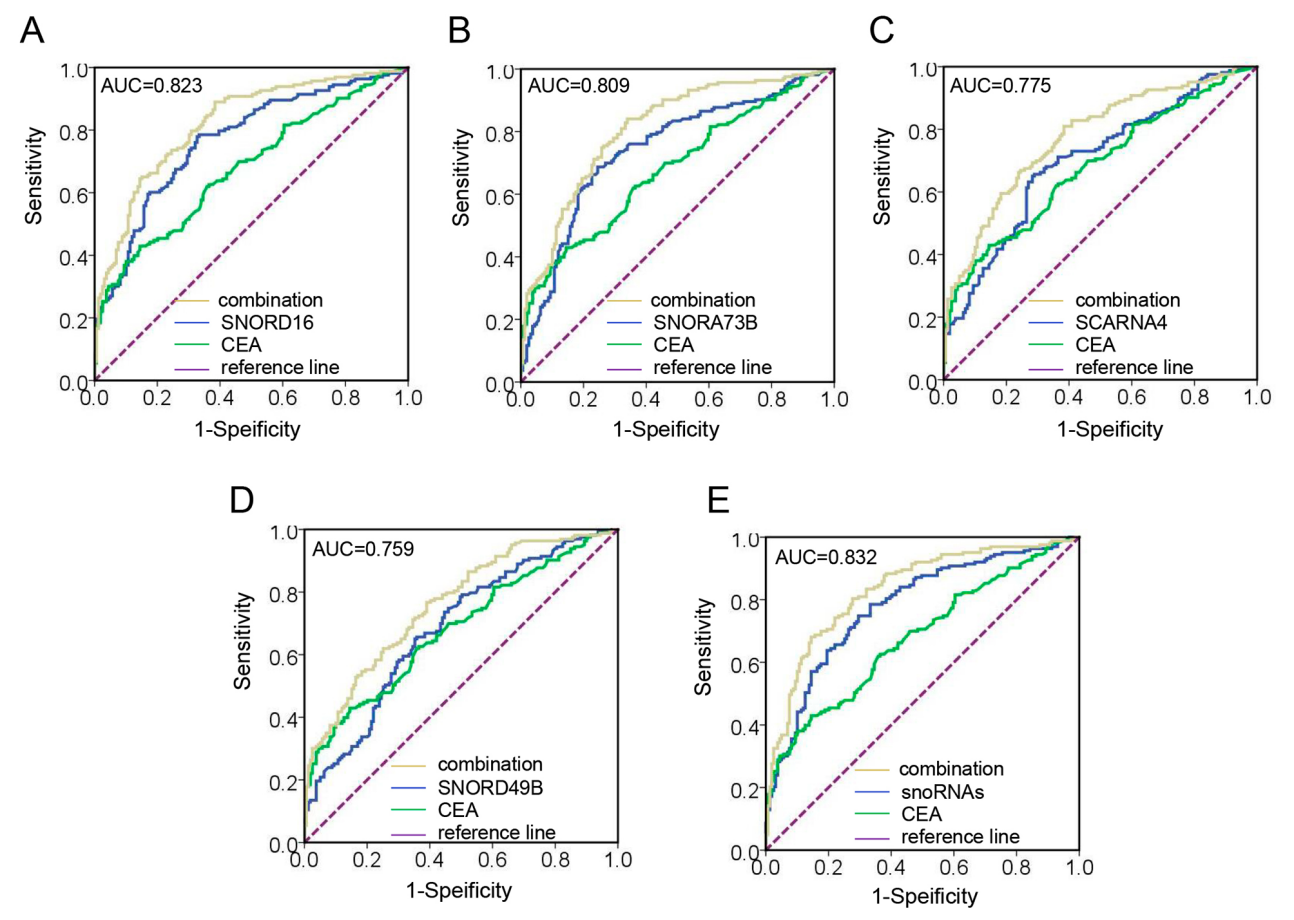
Plasma snoRNAs enhance the diagnostic accuracy of CEA for BC. The AUCs of CEA combined with SNORD16 **(A)**, SNORA73B **(B)**, SCARNA4 **(C)**, SNORD49B **(D)**, and their combination **(E)** in BC patients relative to healthy individuals

Carcinoembryonic antigen (CEA), a polysaccharide-protein complex, is mostly present in the intestinal mucosa of developing embryos as well as tissues from colon and rectal cancer. As a broad-spectrum tumor marker, CEA is closely related to the onset of malignancies such as breast cancer [24, 25]. Thus, we examined the diagnostic performance of our four snoRNAs in conjunction with CEA. Unexpectedly, as shown in Fig. 5A-E, the combination of CEA with plasma snoRNAs significantly improved the diagnostic efficiency compared to CEA alone in BC. The AUC of SNORD16 increased from 0.768 to 0.823, the AUC of SNORA73B increased from 0.744 to 0.809, the AUC of SCARNA4 increased from 0.700 to 0.775, and the AUC of SNORD49B increased from 0.683 to 0.759. Moreover, when combined with the four snoRNAs, the AUC increased to 0.832 with a sensitivity of 68.71% and a specificity of 84.91% (95% CI, 0.785–0.896).

**Fig. 6:**
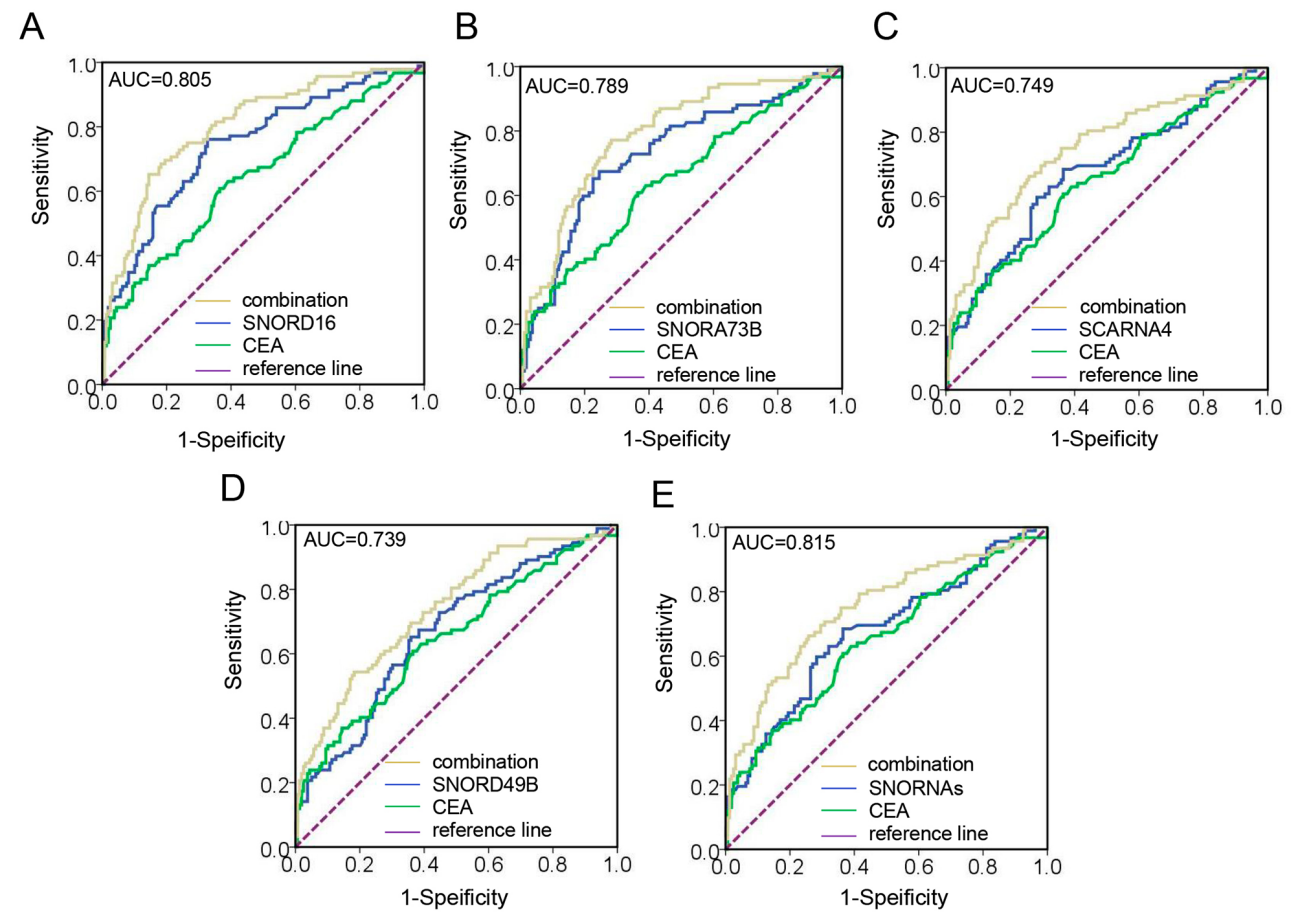
Plasma snoRNAs enhance the early diagnostic accuracy of CEA for BC. The AUCs of CEA combined with SNORD16 **(A)**, SNORA73B **(B)**, SCARNA4 **(C)**, SNORD49B **(D)**, and their combination **(E)** in early-stage BC patients relative to healthy individuals

At the same time, in early-stage BC (Fig. 6A-E), the AUC of SNORD16 combined CEA increased from 0.747 to 0.805, the AUC of SNORA73B increased from 0.730 to 0.789, the AUC of SCARNA4 increased from 0.674 to 0.749, and the AUC of SNORD49B increased from 0.668 to 0.739. Similarly, when combined with the four snoRNAs, the AUC increased to 0.815 with a sensitivity of 76.09% and a specificity of 67.3% (95% CI, 0.785–0.896). Taken together, plasma snoRNAs can increase the diagnostic efficacy of established CEA and BC, as well as their early stages.

## Discussion

Unlike other non-coding RNA, snoRNAs are truly advantageous to act as cancer biomarkers. SnoRNAs are composed of conserved box regions binding with proteins to form stable and functional snoRNPs and of a unique 10–20 nt sequence, which provides high specificity for each snoRNA [4]. These proteins contribute significantly to the processing stability of snoRNAs, allowing them to be stably expressed and detectable in body fluids, including blood plasma, serum, urine, and other body fluids [7]. Moreover, aberrant expression of snoRNAs is prevalent in various cancers; some of these anomalies are cancer-type-specific and closely associated with diagnosis, prognosis, and subtype classification [7]. Given these specifics, snoRNAs are therefore potential candidates as cancer biomarkers. In the current study, we demonstrated that plasma snoRNAs: SNORD16, SNORA73B, SCARNA4, and SNORD49B exerted the capability to act as non-invasive biomarkers for BC.

Previous studies have shown that snoRNA expression can be used for the diagnosis of various cancer types [26], but only few studies have reported the diagnostic role of plasma snoRNAs in BC. In the current study, we confirmed that four snoRNAs (SNORD16, SNORA73B, SCARNA4, and SNORD49B) acted as novel diagnostic biomarkers for BC. First of all, we found four snoRNAs: SNORD16, SNORA73B, SCARNA4, and SNORD49B increased in the plasma of patients with breast cancer, as well as in patients with early-stage. Secondly, these four snoRNAs possessed quite high diagnostic efficiency for diagnosis and early diagnosis of BC, indicating the potential as circulating biomarkers. Besides, we also confirmed that plasma snoRNAs enhance the diagnostic accuracy of CEA for BC; all of them increased the AUC of CEA individually or combinedly. In addition, we found that SNORD16 as well as SNORA73B exerted more effective efficiency than the other two, not only for BC diagnostics but also for the early diagnostics. They were the most contributors to the combined diagnosis of BC; the sensitivity and specificity of AUC combined with SNORD16 or SNORA73B seemed only slightly higher than those of it used individually, indicating SNORD16 and SNORA73B might play a similar but not synergistical role in the diagnosis of BC.

SNORD16, previously named U16 in Homo sapiens, is a 101 nt gene located on chromosome 15q22.31 and derived from the intronic region of Ribosomal Protein L4 (RPL4). SNORD16 contains a complementary sequence with 18 S rRNA, thereby, it was believed to regulate the biological function of ribosomes. A previous study demonstrated that overexpressing SNORD16 was associated with a worse outcome in patients with colorectal cancer. Additionally, it could encourage the expansion, migration, invasion, and proliferation by preventing apoptosis [27]. SNORA73 belongs to the snoRNA H/ACA box located on chromosome 1p35.3 with a length of 205 nt. SNORA73 can guide dyskerin pseudouridine synthase 1 (DKC1) to target mRNA and cause its pseudo-uridylation. Besides, SNORA73B also affects the alternative splicing of regulator of the chromosome condensation 1 (RCC1), increasing the number of transcripts of RCC1-T2 and RCC1-T3, thus promoting cell proliferation and migration [28]. Few investigations on SCARNA4 and SNORD49B have been conducted yet, and there were no pertinent studies. We also conducted pathway enrichment analysis on the selected genes; they were primarily enriched in the signal pathways associated with PI3K-AKT and cell adhesion (Fig. S1 A-D), closely related to the initiation and spread of cancers. Therefore, we believe that SNORD16, SNORD73B, SCARNA4, and SNORD49B are significant contributors to the development and spread of cancer, even if additional study is required to elucidate their potential molecular pathways.

Several limitations should be carefully considered in the present study. First, the sample size enrolled in the current study was small and implausible for the development and clinical application of these biomarkers. Meanwhile, the follow-up information and other traditional tumor marker information, such as CA125 and CA153, from healthy donors were missing, so we failed to analyze the prognostic values and the combined diagnostic efficacy of these four snoRNAs. Besides, we only evaluated the diagnostic values, but little was involved in their underly mechanisms, thereby an in-depth study on the mechanism based on snoRNA should be further pursued.

## Conclusion and perspective

Minimal invasion, easy method of detection, low cost, and convenient census are the advantages of circulating biomarkers. As described above, snoRNAs were stably present and reliably detectable in circulation; Abnormal snoRNA levels could be useful for diagnosis and prognostic applications in BC. Nevertheless, it’s not fully understood how snoRNAs facilitate BC cells to acquire cancer hallmarks and contribute to therapeutic sensitivity or resistance. Besides, the cell signaling pathways, molecular mechanisms, and their regulation are not very clear and hence require detailed investigation. Another critical point for snoRNA clinical implementation would be to perform clinical utility studies. A well-powered, blinded, and population-targeted prospective clinical trial should be incorporated into further planning. Collectively, we screened out SNORD16, SNORA73B, SCARNA4, and SNORD49B and verified their stable increases in plasma from BC patients, as well as revealed their diagnostic efficiency for BC and early-stage BC, indicating their increased expression can be used as promising noninvasive diagnostic biomarkers for BC.

### Electronic supplementary material

Below is the link to the electronic supplementary material.

Supplementary Material 1: Figure S1: Pathway enrichment analysis of SNORD16 **(A)**, SNORA73B **(B)**, SCARNA4 **(C)**, and SNORD49B **(D)**.

Supplementary Material 2: Figure S2: The differential expressions of plasma snoRNAs in a small-size cohort. The differential expressions of plasma SNORD19B, SNORA25, SNORA65, SNORD15A, SNORD2, SNORD12B, SNORA64, SNORA70B, SNORA24, U76, U44, SNORD5, SNORA69, SNORD41, SNORD16, SNORA73B, SCARNA4, and SNORD49B were analyzed in BC patients compared with health donors. ****P<0.0001, ***P<0.001, **P<0.005, ns: no significance.

Supplementary Material 3: Table S1: Cases of healthy volunteers (HD) and breast cancer patients (BC)

## Data Availability

The datasets used and analyzed during the current study are available from the corresponding author upon reasonable request.

## Abbreviations

BC: Breast cancer
snoRNAs: small nucleolar RNAs
nt: nucleotides
TCGA: The Cancer Genome Atlas
cDNA: complementary DNA
SPSS: Statistical Product and Service Solutions
ROC: Receiver Operator Characteristic
AUC: Area under the curve
ns: no significance
HD: Healthy donors
MV: Microvesicles
