# Supplementary figures and images for: Identification of four snoRNAs (SNORD16, SNORA73B, SCARNA4, and SNORD49B) as novel non-invasive biomarkers for diagnosis of breast cancer
Source: Cancer Cell Int. 2024 Feb 4;24:55. doi: 10.1186/s12935-024-03237-0 (PMC10840236; doi:10.1186/s12935-024-03237-0)

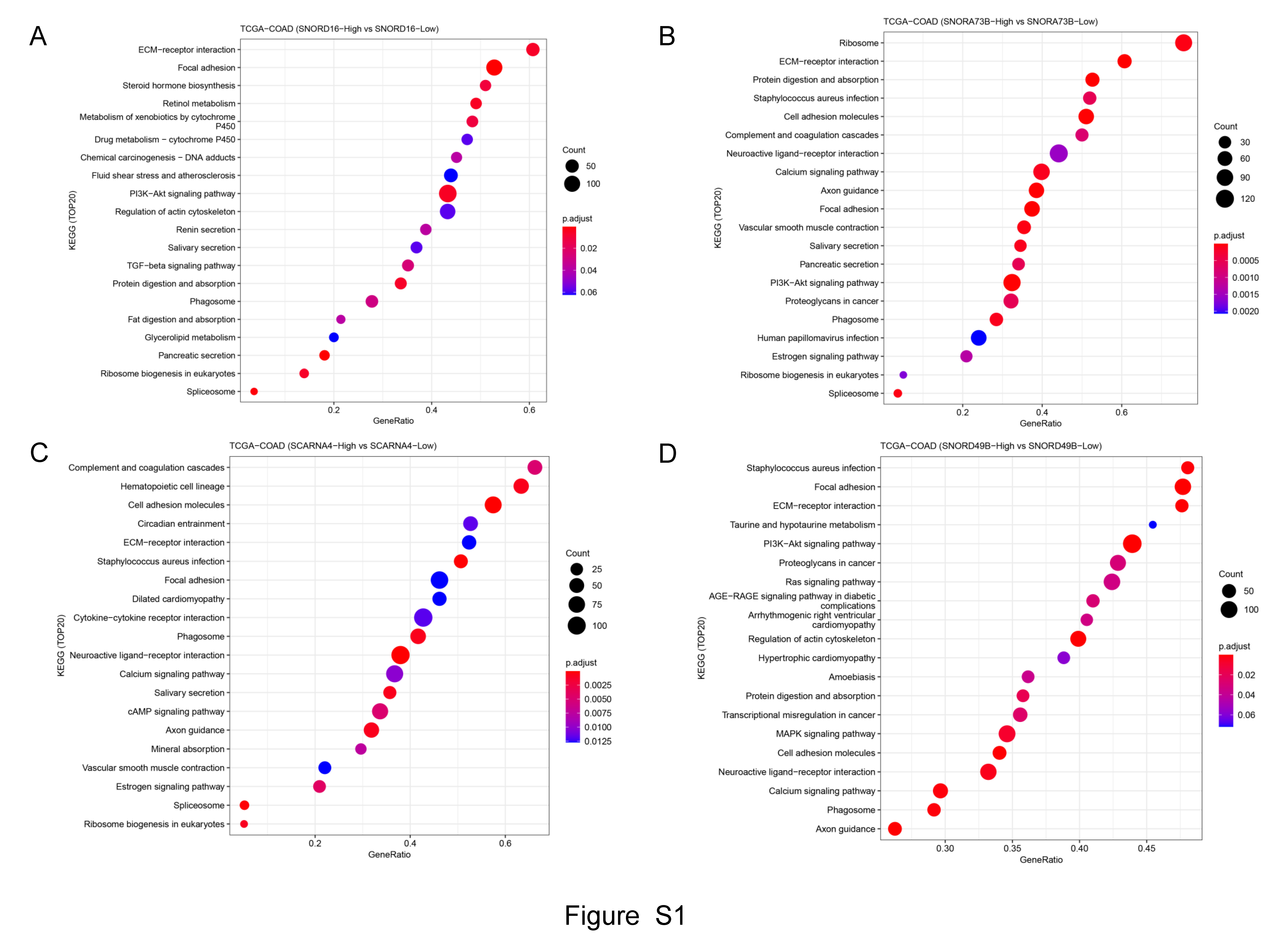

Supplement: Supplementary file 1 — Supplementary Material 1: Figure S1: Pathway enrichment analysis of SNORD16 (A), SNORA73B (B), SCARNA4 (C), and SNORD49B (D). [file 12935_2024_3237_MOESM1_ESM.jpg]

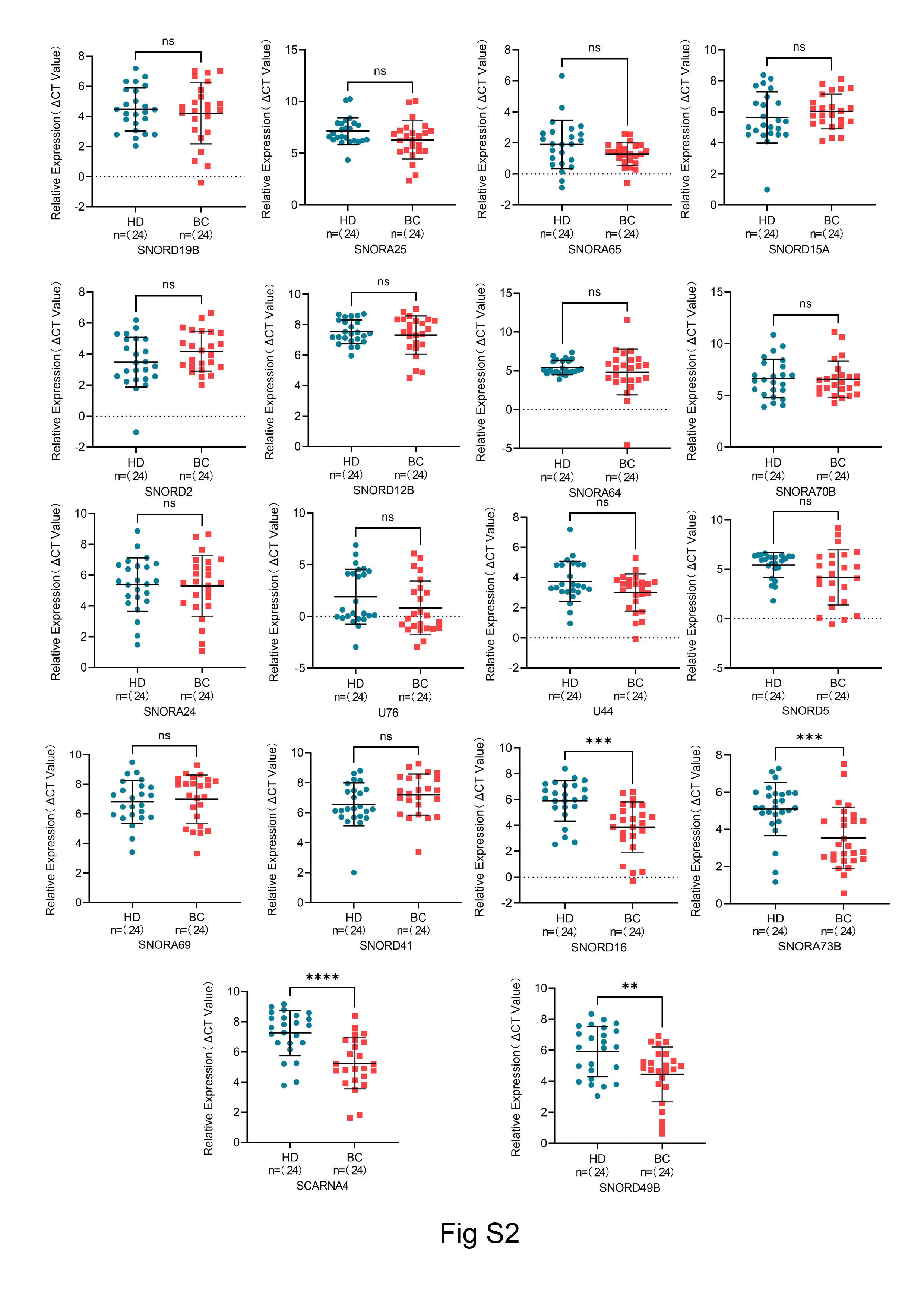

Supplement: Supplementary file 2 — Supplementary Material 2: Figure S2: The differential expressions of plasma snoRNAs in a small-size cohort. The differential expressions of plasma SNORD19B, SNORA25, SNORA65, SNORD15A, SNORD2, SNORD12B, SNORA64, SNORA70B, SNORA24, U76, U44, SNORD5, SNORA69, SNORD41, SNORD16, SNORA73B, SCARNA4, and SNORD49B were analyzed in BC patients compared with health donors. ****P<0.0001, ***P<0.001, **P<0.005, ns: no significance. [file 12935_2024_3237_MOESM2_ESM.jpg]
