# Supplementary material for: Identification of four snoRNAs (SNORD16, SNORA73B, SCARNA4, and SNORD49B) as novel non-invasive biomarkers for diagnosis of breast cancer
Source: Cancer Cell Int. 2024 Feb 4;24:55. doi: 10.1186/s12935-024-03237-0 (PMC10840236; doi:10.1186/s12935-024-03237-0)
Supplement: Supplementary file 3 — Supplementary Material 3: Table S1: Cases of healthy volunteers (HD) and breast cancer patients (BC) [file 12935_2024_3237_MOESM3_ESM.docx]

**Table S1: Cases of healthy volunteers (HD) and breast cancer patients (BC)**

| Gene | HD | BC | early-stage BC |
| --- | --- | --- | --- |
| SNORD16 | 221 | 228 | 126 |
| SNORA73B | 236 | 234 | 127 |
| SCARNA4 | 225 | 233 | 125 |
| SNORD49B | 225 | 230 | 125 |
| Shared | 203 | 212 | 119 |
| Combined with CEA | 159 | 163 | 92 |
